# Health Care Perceptions and a Concierge-Based Transplant Evaluation for Patients With Kidney Disease

**DOI:** 10.1001/jamanetworkopen.2024.47335

**Published:** 2024-11-26

**Authors:** Miriam Vélez-Bermúdez, Yuridia Leyva, Jamie M. Loor, Mary Amanda Dew, Yiliang Zhu, Mark L. Unruh, L. Ebony Boulware, Amit Tevar, Larissa Myaskovsky

**Affiliations:** 1 Center for Healthcare Equity in Kidney Disease, Office of Research, Health Sciences Center, The University of New Mexico, Albuquerque; 2 Department of Psychiatry, University of Pittsburgh, Pittsburgh, Pennsylvania; 3 Department of Internal Medicine, School of Medicine, Health Sciences Center, The University of New Mexico, Albuquerque; 4 Division of Nephrology, Department of Internal Medicine, School of Medicine, Health Sciences Center, The University of New Mexico, Albuquerque; 5 School of Medicine, Wake Forest University, Winston-Salem, North Carolina; 6 Department of Surgery, University of Pittsburgh, Pittsburgh, Pennsylvania

## Abstract

**Importance:**

The kidney transplant (KT) evaluation process is particularly time consuming and burdensome for Black patients, who report more discrimination, racism, and mistrust in health care than White patients. Whether alleviating patient burden in the KT evaluation process may improve perceptions of health care and enhance patients’ experiences is important to understand.

**Objective:**

To investigate whether Black and White participants would experience improvements in perceptions of health care after undergoing a streamlined, concierge-based approach to KT evaluation.

**Design, Setting, and Participants:**

This prospective cohort study from a single urban transplant center included Black and White English-speaking adults who were referred for KT and deemed eligible to proceed with the KT evaluation process. The patients responded to baseline and follow-up questionnaires. The study was conducted from May 2015 to June 2018. Questionnaires were collected before KT evaluation initiation (baseline) and after KT evaluation completion (follow-up). Data were analyzed from October 2022 to January 2024.

**Exposure:**

Data were stratified by race (Black compared with White) and time (baseline compared with follow-up).

**Main Outcomes and Measures:**

The main outcomes were experiences of discrimination in health care, perceived racism in health care, medical mistrust of health care systems, and trust in physician. Repeated-measures regression was used to assess race, time, and the race-by-time interaction as factors associated with each outcome.

**Results:**

The study included 820 participants (mean [SD] age, 56.50 [12.93] years; 514 [63%] male), of whom 205 (25%) were Black and 615 (75%) were White. At baseline and follow-up, Black participants reported higher discrimination (119 [58%]; χ^2^_1_ = 121.89; *P* < .001 and 77 [38%]; χ^2^_1_ = 96.09; *P* < .001, respectively), racism (mean [SD], 2.73 [0.91]; *t*_290.46_ = 7.77; *P* < .001 and mean [SD], 2.63 [0.85]; *t*_296.90_ = 7.52; *P* < .001, respectively), and mistrust (mean [SD], 3.32 [0.68]; *t*_816.00_ = 7.29; *P* < .001 and mean [SD], 3.18 [0.71]; *t*_805.00_ = 6.43; *P* < .001, respectively) scores but lower trust in physician scores (mean [SD], 3.93 [0.65]; *t*_818.00_ = −2.01; *P* = .04 and mean [SD], 3.78 [0.65]; *t*_811.00_ = −5.42; *P* < .001, respectively) compared with White participants. All participants experienced statistically significant reductions in discrimination (Black participants: odds ratio, 0.27 [95% CI, 0.16-0.45]; *P* < .001; White participants: odds ratio, 0.37 [95% CI, 0.25-0.55]; *P* < .001) and medical mistrust in health care (Black participants: β [SE], −0.16 [0.05]; *P* < .001; White participants: β [SE], −0.09 [0.03]; *P* < .001), and Black participants reported lower perceived racism at follow-up (β [SE], −0.11 [0.05]; *P* = .04). There was a statistically significant race-by-time interaction outcome in which Black participants’ trust in physicians was significantly lower at follow-up, but White participants reported no change.

**Conclusions and Relevance:**

The findings of this cohort study of patients who underwent a streamlined, concierge-based KT evaluation process suggest that a streamlined approach to clinic-level procedures may improve patients’ perceptions of the health care system but may not improve their trust in physicians. Future research should determine whether these factors are associated with KT outcome, type of KT received, and time to KT.

## Key Points

**Question:**

Can negative perceptions of health care change among kidney transplant candidates who undergo a streamlined, concierge-based approach to kidney transplant evaluation?

**Findings:**

In this cohort study of 820 individuals who underwent a streamlined approach to kidney transplant evaluation, participants reported less discrimination and medical mistrust in health care overall, and Black participants reported less perceived racism in health care. However, Black participants reported less physician trust at follow-up, while White participants reported no change.

**Meaning:**

These findings suggest that adopting approaches that streamline clinic-level procedures may improve patient perceptions of health care among kidney transplant candidates.

## Teaser

This cohort study investigates whether Black and White participants experience improvements in perceptions of health care after undergoing a streamlined, concierge-based approach to kidney transplant evaluation.

## Introduction

Kidney transplantation is the optimal kidney replacement therapy for kidney failure.^1,2,3^ The kidney transplant (KT) evaluation process to determine eligibility for KT is complex and lengthy, requiring patients to attend a full day of evaluation at a transplant center.^4,5,6,7,8,9,10,11^ If patients are deemed potentially eligible for transplant at this initial visit, they must complete multiple clinical tests before the transplant team can determine transplant eligibility.^10,11^ Often, the patient is largely responsible for managing and navigating these numerous clinic appointments across various specialties and health care professionals, which can be challenging.^5,6,7,8,9,10,11^

Evidence suggests that this process is longer for Black patients compared with non-Hispanic White patients,^5,6,7,8,9^ and Black patients face more barriers at every step when pursuing KT. They are less likely to complete the KT evaluation due to delayed referrals,^5,7,9,10^ spend more time on KT waiting lists,^6,8^ and are less likely to undergo a transplant compared with White patients,^1,3^ despite a higher prevalence of kidney failure.^3^ Myaskovsky et al^7^ found that Black patients took longer to complete the KT evaluation process compared with White patients. Furthermore, Black patients reported more discrimination and racism in health care and more medical mistrust in health care systems compared with White patients.^7^

Several studies have examined the experience of racial discrimination in health care when interacting with health care professionals across various clinical contexts, including but not limited to diabetes, hypertension, and chronic kidney disease.^12,13,14,15,16,17,18,19,20,21,22^ Scoping reviews and meta-analyses indicate that racial and ethnic minority patients consistently report greater discrimination and racism in health care compared with non-Hispanic White patients in the US.^12,13^ According to prior qualitative work with Black patients, self-reported racism in health care manifests as perceived exclusion from health care–related decision-making processes, differential care due to race, poor patient-physician interaction, and thus mistrust of health care professionals’ clinical recommendations.^14,15,16,17^ Survey-based research identified associations between racial discrimination in health care and greater mistrust in health care professionals and systems,^18,19^ which is also associated with delayed or unmet care.^20,21,22^ In 1 study, negative experiences with health care were associated with a lower likelihood of initiating the KT evaluation.^21^ These studies often collect data cross-sectionally, making it difficult to discern the temporal association between negative health care experiences and negative health care perceptions. Therefore, it is unclear whether improving health care experiences might be associated with an improvement in self-reported perceptions of health care.

Within the context of KT, alleviating patient burden associated with managing the KT evaluation process may change patients’ perceptions regarding health care and improve their overall experience. Myaskovsky et al^23,24^ worked with a large, single-center urban KT clinic and its hospital administration to implement the Kidney Transplant Fast Track (KTFT) program, which streamlined the KT evaluation process by providing a concierge-based approach to scheduling all patients’ appointments and coordinating the receipt of pretransplant testing. Following patients’ initial KT evaluation appointment, a transplant clinic coordinator arranged and scheduled all clinic tests for each patient rather than providing patients with a list of tests to be completed on their own.^23,24^

Apart from the KTFT’s primary aim of streamlining the evaluation process,^23,24^ we had an opportunity to assess whether secondary outcomes were observed, such as changes in self-reported experiences and perceptions of health care, after patients underwent a concierge-based approach to KT evaluation.^25^ In this prospective cohort study of candidates for KT, all of whom underwent a streamlined approach to KT evaluation, we aimed to assess whether receiving the services offered by the KTFT was associated with subsequent improvements in perceptions of health care at follow-up. We had 3 major hypotheses. First, we expected that all participants would report better perceptions of health care after undergoing the KTFT because the program would minimize the burden of scheduling a clinical work-up for the KT evaluation.^23^ Second, we hypothesized that Black participants would endorse more negative experiences and perceptions of health care overall compared with White participants based on evidence from past studies.^7,12,13,26,27,28^ However, for our third hypothesis, we expected that the observed changes in experiences and perceptions of health care at follow-up would be greater among Black participants compared with White participants.

Because race is often used as a proxy for a broad range of social constructs,^7,29,30^ we examined the potential association of several sociodemographic and psychosocial factors in our study. Additionally, race has historically been used to erroneously account for differences in kidney functioning^31^ when specific medical factors should have been included as critical covariates instead, as we did in our analyses. We also adjusted for transplant-related knowledge and concerns because they have been previously associated with clinic-related outcomes in patients undergoing KT.^7,32,33^ Finally, we accounted for participants’ KT waiting list status at follow-up and time to complete the KT evaluation process from the initial visit, as we believed these factors may also inform participants’ reporting of experiences and perceptions of health care.

## Methods

### Study Sample and Procedures

**Figure 1.**
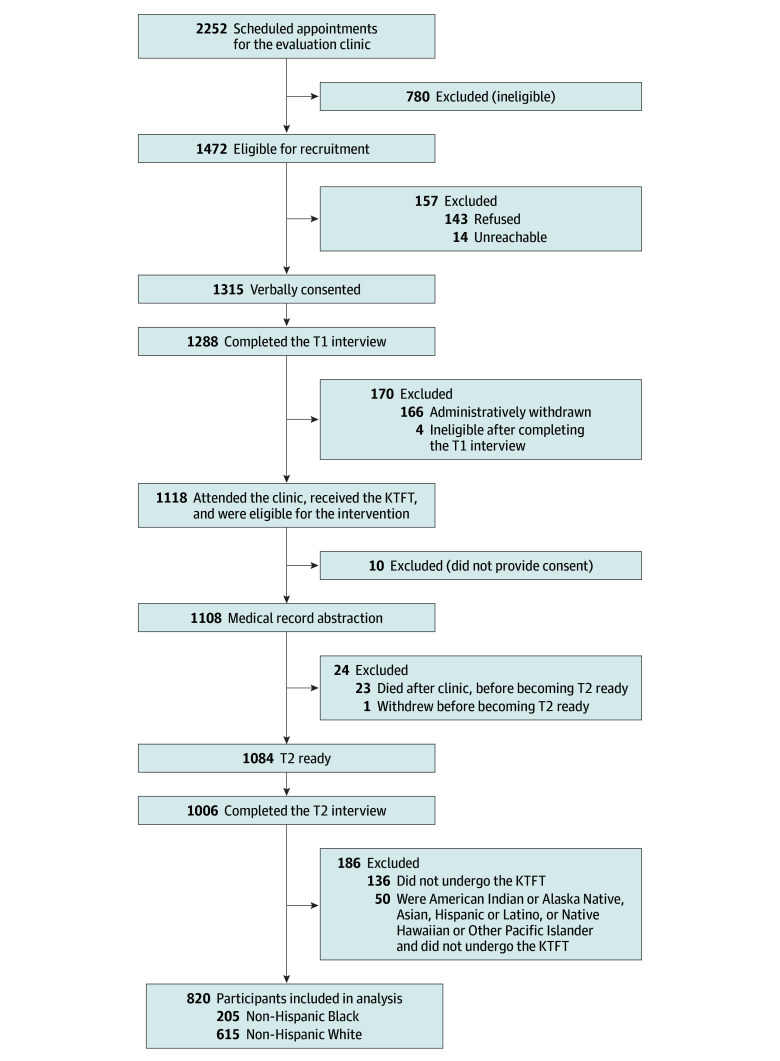
Cohort Recruitment Efforts and Sample Size Flowchart KTFT indicates Kidney Transplant Fast Track; T1, baseline interview; T2, follow-up interview.

The sample for this cohort study came from a larger experimental trial (Increasing Equity in Transplant Evaluation and Living Donor Kidney Transplantation^23^) that included a prospective cohort of patients undergoing initial evaluation for KT at a single center between May 2015 and June 2018; the patients also received the KTFT (the study protocol can be found in Bornemann et al^23^; the patient flow diagram is provided in Figure 1). This subsample had comparable age, sex, and racial characteristics to the larger study’s cohort but included a slightly higher proportion of Black patients and males compared with the overall clinic population of the larger study’s cohort during the time frame. The study followed the Strengthening the Reporting of Observational Studies in Epidemiology (STROBE) reporting guideline. The institutional review boards at the University of Pittsburgh and the University of New Mexico approved this study, and a data use agreement was signed between the 2 institutions. Written informed consent was obtained from all participants. The study was conducted in accordance with the Declaration of Helsinki^34^ and is consistent with the principles of the Declaration of Istanbul as outlined in the Declaration of Istanbul on Organ Trafficking and Transplant Tourism.^35^

Patients were eligible for the parent study if they were 18 years or older, English speaking, referred for KT, had not previously undergone KT, did not have cognitive or sensory impairments that prevented them from participating, and were deemed eligible to proceed with the KT evaluation process by the transplant team at their initial appointment. We recruited patients after they scheduled their initial KT evaluation appointment.

We used telephone interviews and paper surveys to collect survey responses at baseline (before the initial KT evaluation) and at follow-up (after the evaluation process ended) whether they successfully completed the evaluation or were withdrawn. The median time from evaluation completion to follow-up was 33 days (IQR, 7-69 days).

For this secondary study, inclusion criteria from the parent study were limited to participants who self-identified as either non-Hispanic Black (hereinafter, Black) or non-Hispanic White (hereinafter, White) and excluded all other individuals who self-identified as any other racial or ethnic category (ie, American Indian or Alaska Native, Asian, Hispanic or Latino, or Native Hawaiian or Other Pacific Islander). These data were collected because the primary aim of the parent study was to reduce racial disparities associated with the KT evaluation period. We also excluded participants who were rejected for KT at the initial KT evaluation and therefore did not undergo the KT evaluation process.

### Variables

Surveys collected self-reported demographic characteristics (eg, age, race), transplant knowledge and concerns, and psychosocial factors at baseline. Medical factors were abstracted from electronic medical records. All variables were considered for inclusion in our analyses. eTable 1 in Supplement 1 provides a full description of the variables.

We assessed perceptions of health care with the following 4 previously validated measures: (1) experience of discrimination in health care (ie, discrimination; reporting of personal experiences of discrimination during interactions with health care professionals, 7 items, and response range: never to always),^26,36^ (2) perceived racism in health care (ie, racism; reporting the extent to which racism is common in health care, 4 items, and response range: strongly disagree to strongly agree),^27^ (3) medical mistrust of the health care system (ie, mistrust; reporting the extent to which hospital systems are untrustworthy, incompetent, and not acting in patients’ best interest, 7 items, and response range: strongly disagree to strongly agree),^27,37,38^ and (4) trust in physician (ie, reporting the extent to which respondents trust their physicians, 11 items, response range: totally disagree to totally agree).^39^ Each measure used a 5-point Likert scale. Items were averaged to create scale scores from 1 to 5, with higher scores reflecting greater discrimination, racism, medical mistrust, and trust in physician. Responses were collected at baseline and at follow-up (eTable 1 in Supplement 1).

### Statistical Analysis

Data were analyzed from October 2022 to January 2024. We examined data for missingness and whether assumptions (eg, normality, outliers) for analyses were met, and we adjusted accordingly. We confirmed minimal missing data with no evidence of any systematic pattern of missingness, thus suggesting that an assumption of missingness at random was appropriate for our data. We calculated frequencies and percentages for categorical variables and means and SDs for continuous variables. We compared Black and White participants on all survey items (Table 1).

**Table 1.** Characteristics by Total Cohort and Race^a^

Characteristic	Participant group			Test statistic^b^	*P* value
	Total (N = 820)	Black (n = 205)	White (n = 615)		
**Demographic characteristics**					
Sex					
Female	306 (37)	76 (37)	230 (37)	χ^2^_1_ = 0.01	.93
Male	514 (63)	129 (63)	385 (63)		
Age at baseline completion, mean (SD), y	56.50 (12.93)	53.76 (13.28)	57.61 (12.69)	*t*_818.00_ = −4.31	<.001
≤12th Grade education	366 (45)	100 (49)	266 (43)	χ^2^_1_ = 1.90	.17
<$50 000 Family income	557 (70)	170 (87)	387 (65)	χ^2^_1_ = 33.89	<.001
Insurance status					
Public only	338 (41)	136 (66)	202 (33)	χ^2^_2_ = 71.66	<.001
Private only	167 (20)	27 (13)	140 (23)		
Public and private	315 (38)	42 (20)	273 (44)		
Part-time or full-time employment status	221 (27)	46 (22)	175 (28)	χ^2^_1_ = 2.83	.09
Married or domestic partnership	416 (51)	62 (30)	342 (56)	χ^2^_1_ = 39.58	<.001
No. of people in one’s social network, mean (SD)^c^	25.01 (19.68)	27.15 (20.65)	24.30 (19.31)	*t*_818.00_ = 1.80	.07
**Transplant knowledge and concerns**					
Transplant knowledge, mean (SD)	11.06 (2.83)	10.15 (2.93)	11.36 (2.73)	*t*_818.00_ = −5.43	<.001
No. of learning activities, mean (SD)	2.22 (1.11)	1.96 (1.07)	2.30 (1.11)	*t*_818.00_ = −3.98	<.001
Learning activities, h					
0-2	261 (32)	79 (39)	182 (30)	χ^2^_2_ = 6.80	.03
>2-5	216 (26)	54 (26)	162 (26)		
>5	343 (42)	72 (35)	271 (44)		
Transplant concerns, mean (SD)	43.68 (7.56)	45.10 (8.09)	43.20 (7.32)	*t*_818.00_ = 3.12	.002
**Medical factors**					
Dialysis type					
Hemodialysis	398 (49)	135 (66)	263 (43)	χ^2^_2_ = 40.45	<.001
Peritoneal dialysis	90 (11)	25 (12)	65 (11)		
No dialysis	332 (40)	45 (22)	287 (47)		
Dialysis duration, y					
Never on dialysis	318 (39)	41 (20)	277 (45)	χ^2^_3_ = 82.66	<.001
<1	304 (37)	85 (41)	219 (36)		
1-5	158 (19)	49 (24)	109 (18)		
>5	40 (5)	30 (15)	10 (2)		
Waiting list status at follow-up					
Accepted for KT waiting list	462 (56)	90 (44)	372 (60)	χ^2^_2_ = 19.71	<.001
Rejected for KT waiting list	138 (17)	38 (19)	100 (16)		
Evaluation closed or incomplete	220 (27)	77 (38)	143 (23)		
Time from evaluation to follow-up, mean (SD), d	184.72 (159.56)	228.30 (171.30)	170.20 (152.90)	*t*_319.21_ = 4.32	<.001
Overweight or obesity	646 (79)	158 (77)	488 (79)	χ^2^_1_ = 0.48	.49
Charlson Comorbidity Index score, mean (SD)	4.21 (1.73)	4.51 (1.97)	4.12 (1.63)	*t*_302.90_ = 2.57	.01
**Psychosocial factors**					
Social support, mean (SD)	41.58 (6.41)	40.65 (7.06)	41.89 (6.15)	*t*_313.40_ = −2.24	.03
Anxiety (>moderate)	35 (4)	8 (4)	27 (4)	χ^2^_1_ = 0.09	.76
Depression (>moderate)	42 (5)	15 (7)	27 (4)	χ^2^_1_ = 2.71	.10
Health literacy, mean (SD)	3.83 (1.02)	3.82 (1.02)	3.83 (1.02)	*t*_818.00_ = −0.12	.90
Family loyalty, mean (SD)^d^	3.18 (0.60)	3.34 (0.64)	3.12 (0.57)	*t*_817.00_ = 4.55	<.001
Any vs no religious objection to living-donor KT^e^	401 (49)	87 (43)	314 (52)	χ^2^_1_ = 4.37	.04

Abbreviation: KT, kidney transplant.

^a^ Data are presented as No. (%) unless otherwise indicated. All variables reflect scores and values at baseline except for waiting list status at follow-up and time from evaluation to follow-up. The following score ranges are for continuous variables derived from validated measures at baseline, with higher scores indicating more of that variable (eg, more social support): transplant knowledge: 0-19, transplant concerns: 0-60, social support: 12-48, and family loyalty: 1-5.

^b^ Calculations for *χ*^2^ were conducted for categorical variables by race, and *t* tests were conducted for continuous variables by race.

^c^ Indicates potential living donors.

^d^ Data were missing for 1 participant.

^e^ Data were missing for 9 participants.

We examined normality and symmetry assumptions for each of the 4 outcomes and found them satisfactory except for the discrimination measure. Prior literature recommended dichotomizing the discrimination measure into ever and never experienced discrimination because the original measure (never to always) typically yielded skewed data,^26^ as it did in our sample. We compared Black and White participants using χ^2^ for the discrimination measure and using *t* tests for the racism, medical mistrust, and trust in physician measures. We compared participants at baseline and follow-up (Table 2). There were very few instances of missing data, with no apparent differences by waiting list status at follow-up (eTable 2 in Supplement 1).

**Table 2.** Unadjusted Experience of Discrimination, Perceived Racism, Medical Mistrust, and Trust in Physician at Baseline and Follow-Up

Outcome	Baseline					Follow-up				
	Participant group			Test statistic^a^	*P* value	Participant group			Test statistic^a^	*P* value
	Total (N = 820)	Black (n = 205)	White (n = 615)			Total (N = 820)	Black (n = 205)	White (n = 615)		
Experiences of discrimination, No. (%)^b^	230 (28)	119 (58)	111 (18)	χ^2^_1_ = 121.89	<.001	130 (16)	77 (38)	53 (9)	χ^2^_1_ = 96.09	<.001
Perceived racism, mean (SD)^c^^,^^d^	2.32 (0.80)	2.73 (0.91)	2.19 (0.71)	*t*_290.46_ = 7.77	<.001	2.26 (0.77)	2.63 (0.85)	2.13 (0.69)	*t*_296.90_ = 7.52	<.001
Medical mistrust, mean (SD)^d^^,^^e^	3.00 (0.76)	3.32 (0.68)	2.89 (0.75)	*t*_816.00_ = 7.29	<.001	2.90 (0.74)	3.18 (0.71)	2.80 (0.73)	*t*_805.00_ = 6.43	<.001
Trust in physician, mean (SD)^f^	4.00 (0.61)	3.93 (0.65)	4.03 (0.60)	*t*_818.00_ = −2.01	.04	3.98 (0.63)	3.78 (0.65)	4.05 (0.61)	*t*_811.00_ = −5.42	<.001

^a^ Calculations for *χ*^2^ were conducted for categorical variables by race, and *t* tests were conducted for continuous variables by race. For continuous variables, ranges are from 1 (low) to 5 (high).

^b^ Indicates ever experienced discrimination. Data were missing for 2 participants at follow-up.

^c^ Data were missing for 4 participants at baseline and 15 participants at follow-up.

^d^ Range: 1.00-5.00.

^e^ Data were missing for 2 participants at baseline and 13 participants at follow-up.

^f^ Range: 1.45-5.00. Data were missing for 7 participants at follow-up.

We used repeated measurements of the outcomes at baseline and follow-up, with a random intercept, to test whether time (baseline compared with follow-up) and race (Black compared with White) and the interaction of time by race were associated with each outcome variable. When a random intercept is included, both linear and logistic regression can accommodate repeated measurements of each participant’s response on the outcome variables.^40,41^ This approach allows for all data to be included without deletion of participants under the missingness-at-random assumption.^40,41,42^ We adjusted for baseline demographic, transplant, psychosocial, and medical factors in the models. We conducted multivariable logistic regression for discrimination and multivariable linear regression for racism, medical mistrust, and trust in physician.

In our multivariable regression analyses, we used a hierarchical approach.^43^ Thus, in block 1, we examined main outcomes of race and time to test our first and second hypotheses that responses to outcome variables may improve from baseline to follow-up and that Black participants would endorse more negative experiences and perceptions of health care. Then, in block 2, we added a race-by-time interaction term to test our third hypothesis: that Black participants would experience greater changes in the outcome variables than White participants. To better understand within-race changes from baseline to follow-up, we examined simple outcomes within each group regardless of whether the race-by-time interaction was significant.

To determine which covariates would be included in multivariable repeated-measures regression models, we examined a series of univariable analyses with each of the 4 outcome variables. If a factor was associated with any of the 4 outcome variables at baseline, it was included in all subsequent analyses. This liberal inclusion criteria prevented any relevant variables from being inadvertently excluded. All demographic, transplant factors, medical, and psychosocial characteristics were tested for inclusion in multivariable modeling. Two-sided *P* < .05 was considered statistically significant. Data analyses were performed using SAS Maintenance 8, version 9.4M8 (SAS Institute Inc).

## Results

### Sample Characteristics

A total of 820 participants were included in the study (mean [SD] age, 56.50 [12.93] years; 306 [37%] female and 514 [63%] male), of whom 205 (25%) were Black and 615 (75%) were White (Table 1). Black and White participants significantly differed on several demographic, medical, and psychosocial characteristics. Most factors were included in multivariable modeling based on the aforementioned liberal inclusion criteria. Among all participants, 462 (56%) were accepted for KT at follow-up, and the rest were either rejected for wait-listing (138 [17%]) or their evaluation was closed or incomplete (220 [27%]) (Table 1).

Descriptive statistics (Table 2 and eTable 3 in Supplement 1) indicate that Black participants were statistically more likely to report experiencing discrimination at baseline (119 [58%]; χ^2^_1_ = 121.89; *P* < .001) and at follow-up (77 [38%]; χ^2^_1_ = 96.09; *P* < .001) compared with White participants (at baseline: 111 [18%]; at follow-up: 53 [9%]) (Table 2). Black participants also reported higher racism (at baseline: mean [SD], 2.73 [0.91]; *t*_290.46_ = 7.77; *P* < .001; at follow-up: mean [SD], 2.63 [0.85]; *t*_296.90_ = 7.52; *P* < .001) than White participants (at baseline: mean [SD], 2.19 [0.71]; at follow-up: mean [SD], 2.13 [0.69]) and higher mistrust (at baseline: mean [SD], 3.32 [0.68]; *t*_816.00_ = 7.29; *P* < .001; at follow-up: mean [SD], 3.18 [0.71]; *t*_805.00_ = 6.43; *P* < .001) than White participants (at baseline: mean [SD], 2.89 [0.75]; at follow-up: mean [SD], 2.80 [0.73]) but reported lower trust in physician scores (at baseline: mean [SD], 3.93 [0.65]; *t*_818.00_ = −2.01; *P* = .04; at follow-up: mean [SD], 3.78 [0.65]; *t*_811.00_ = −5.42; *P* < .001) than White participants (at baseline: mean [SD], 4.03 [0.60]; at follow-up: mean [SD], 4.05 [0.61]).

### Multivariable Regression

#### Main Outcomes

We found greater odds of Black participants reporting discrimination (odds ratio [OR], 8.94 [95% CI, 5.60-14.27]; *P* < .001), and they were more likely to report racism (β [SE], 0.55 [0.06]; *P* < .001) and mistrust (β [SE], 0.38 [0.06]; *P* < .001), as well as lower trust in physicians (β [SE], −0.13 [0.05]; *P* = .004) compared with White participants averaged across both time points. Among all participants, there were lower odds of reporting discrimination (OR, 0.33 [95% CI, 0.24-0.46]; *P* < .001), a lower level of racism (β [SE], −0.07 [0.03]; *P* = .01), and a lower level of mistrust (β [SE], −0.11 [0.02]; *P* < .001) at follow-up compared with baseline. However, there was no significant change for trust in physicians from baseline to follow-up (Table 3).

**Table 3.** Factors Associated With Perceptions of Health Care by Race and Time With Multivariable Regression Models^a^

Outcome	Experiences of discrimination		Perceived racism		Medical mistrust		Trust in physician	
	OR (95% CI)	*P* value	β (SE)	*P* value	β (SE)	*P* value	β (SE)	*P* value
Main outcomes								
Black compared with White participants^b^	8.94 (5.60-14.27)	<.001	0.55 (0.06)	<.001	0.38 (0.06)	<.001	−0.13 (0.05)	.004
Follow-up compared with baseline^c^	0.33 (0.24-0.46)	<.001	−0.07 (0.03)	.01	−0.11 (0.02)	<.001	−.01 (.02)	.53
Race by time								
Interaction outcome	0.73 (0.39-1.36)	.32	−0.06 (0.06)	.38	−0.07 (0.06)	.22	−0.16 (0.05)	.002
Change outcomes								
Black participants	0.27 (0.16-0.45)	<.001	−0.11 (0.05)	.04	−0.16 (0.05)	<.001	−0.14 (0.05)	.003
White participants	0.37 (0.25-0.55)	<.001	−0.06 (0.03)	.07	−0.09 (0.03)	<.001	0.03 (0.03)	.33

Abbreviation: OR, odds ratio.

^a^ The multivariable regression model, which assessed a race-by-time interaction term, was further adjusted by main outcomes. Parameter estimates were converted into ORs for experiences of discrimination, and standardized estimates (ie, β) were reported for perceived racism, medical mistrust, and trust in physician. All analyses were adjusted by the following covariates: baseline demographic characteristics (age, sex, marital status, income, employment status, insurance type, and network of potential living kidney donors), baseline transplant knowledge and concerns (transplant knowledge, number of learning activities, hours conducting learning activities, and transplant concerns), baseline medical characteristics (dialysis duration and dialysis type), baseline psychosocial and cultural characteristics (social support, anxiety, depression, health literacy, family loyalty, and religious objection to transplant), waiting list status at follow-up, and number of days to index evaluation completion. eTable 4 in Supplement 1 shows the regression coefficients of perceptions of health care scores by each racial group at each time point. eTables 5 to 12 in Supplement 1 show complete output for each statistical model, including all values for covariates.

^b^ White was the reference category.

^c^ Baseline was the reference category.

#### Race by Time

**Figure 2.**
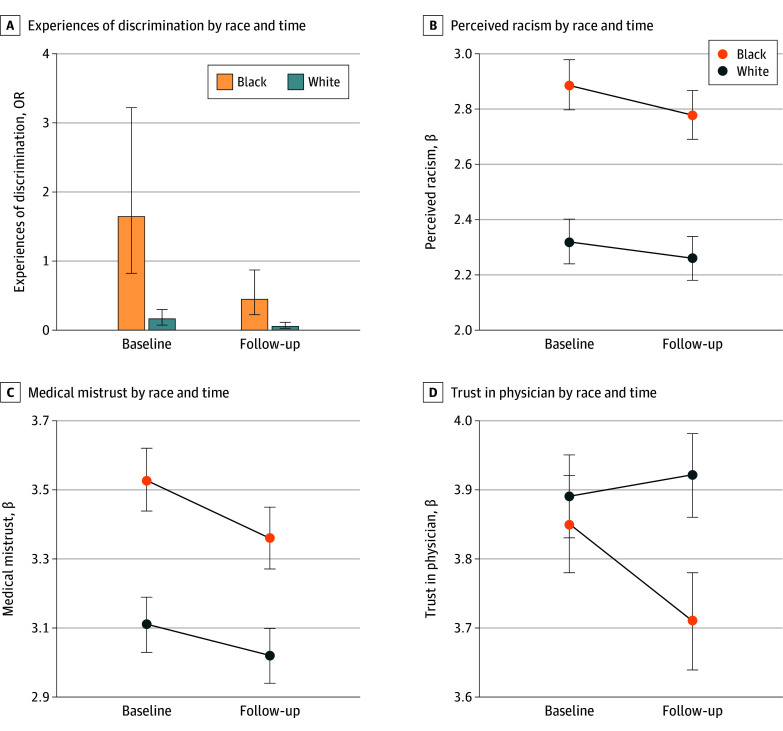
Experiences of Discrimination, Perceived Racism, Medical Mistrust, and Trust in Physician Outcome Variables by Respondent Race (Black Compared With White) and Time (Baseline Compared With Follow-Up) The plots show the pattern of associations between Black and White participants. Error bars indicate 95% CIs (A). Lines reflect standardized estimates (ie, β) from adjusted multivariable linear regression models, with SE values represented in the error bars (B-D). Only trust in physician (D) indicates a significant association with race and time. Least-squares mean and odds ratio (OR) values for these plots are reflected in eTable 4 in Supplement 1.

All participants experienced reductions in discrimination (Black: OR, 0.27 [95% CI, 0.16-0.45]; *P* < .001; White: OR, 0.37 [95% CI, 0.25-0.55]; *P* < .001) and mistrust (Black: β [SE], −0.16 [0.05]; *P* < .001; White: β [SE], −0.09 [0.03]; *P* < .001), and only Black participants reported lower racism (β [SE], −0.11 [0.05]; *P* = .04) at follow-up (Table 3). Contrary to expectations, we did not find that Black participants reported significant differences from baseline to follow-up compared with White participants for these variables (Figure 2A-C). Although we identified a significant change for trust in physician (β [SE], −0.16 [0.05]; *P* = .002), the direction of the outcome was unexpected. Black participants reported significantly lower trust in physician scores at follow-up compared with baseline (β [SE], −0.14 [0.05]; *P* = .003), corresponding to less trust in physicians, but White participants showed no difference in their follow-up compared with baseline scores on this variable (β [SD], 0.03 [0.03]; *P* = .33). Table 3, Figure 2, and eTables 4 to 12 in Supplement 1 provide least-squares mean values and complete output for each model including all covariates.

## Discussion

Many studies have reported an association between health care–related discrimination, racism, and mistrust and health care outcomes.^12,13,14,15,16,17,18,19,20,21,22,44,45,46^ Our prospective cohort study adds to the existing body of primarily cross-sectional research and found that Black and White participants who underwent a concierge-based streamlined approach to KT evaluation reported reductions in discrimination in health care and medical mistrust. Additionally, Black participants reported less perceived racism in health care at follow-up. It was anticipated that ratings of mistrust in hospital systems would improve because the KTFT targeted the organizational level of the KT evaluation process. Similarly, despite the discrimination measure being worded as a lifetime report,^26^ we believe participants may have considered their most recent health care encounters when responding at follow-up. This may explain why they reported fewer experiences of discrimination in health care compared with baseline and why Black participants in particular rated their perceived racism as lower, potentially suggesting that they had a positive experience participating in the KTFT.

We found that Black participants reported a decrease in trust in physician from baseline to follow-up. Although we hypothesized that trust in physician would improve after patients underwent a streamlined KT evaluation process, we found similar patterns previously observed in patients with lung cancer and hypertension and those undergoing surgery after clinical encounters with physicians, unrelated to streamlined health care system processes.^44,45,46^ We suspect that improvements may not have occurred at follow-up^47^ because KTFT targeted transplant clinic procedures rather than the patient-physician encounter. However, we do not know why Black participants reported less trust in physicians at follow-up. It is possible that increased interaction with the health care system may affect trust in physicians, and in this study, the associated positive experience with the concierge-based approach to KT evaluation may have led Black participants to evaluate their physician interactions more critically at follow-up, but future research is warranted.

### Limitations

This study has some limitations. First, all patients received the KTFT, and there was no comparison group of patients undergoing a different approach; thus, we cannot assess whether the streamlined KTFT approach was associated with the changes observed from baseline to follow-up. An alternative explanation is that increased exposure to health care clinics, like being evaluated for KT, is associated with improved perceptions of health care. Additionally, expectations among health care professionals that the KTFT would have been beneficial for patients may have influenced participants’ responses. To our knowledge, however, this is the first study to examine changes in perceptions of health care from baseline to follow-up. To attenuate the influence of any potential biases, we controlled for various baseline factors in analyses and waiting list status at follow-up (eTables 5-12 in Supplement 1). Nevertheless, it is critical for future longitudinal evaluations of perceptions of health care to include a comparison group, ideally in the context of a randomized clinical trial. Second, our sample size of Black participants (25%) was much smaller than our sample of White participants (75%). Although reflective of the patient population where the study took place, we do not know if the results would have changed had the 2 sample sizes been more comparable. Future studies should recruit comparable sample sizes of Black and White patients and patients from other racial and ethnic groups if they are well represented within a clinic. Lastly, the study took place at a single health care center, thus limiting the generalizability.

## Conclusions

In this prospective cohort study of patients evaluated for KT within a clinic that implemented a health care system intervention to shorten the KT evaluation process,^23^ we observed improvements in self-reported experiences and perceptions of health care. These findings were robust even after accounting for potential influencing factors, including whether patients were ultimately wait-listed for KT at follow-up. We observed improvements in health care–related discrimination and mistrust among all participants, regardless of race, and lower reports of perceived racism among Black participants after undergoing a concierge-based approach to streamline KT evaluation. These findings suggest that streamlining processes in care delivery may positively influence patients’ perceptions of health care. The findings also suggest that implementing clinic-level changes may be necessary to observe improvements in perceptions of health care. Because this study focused on the time period during the KT evaluation process, future research should determine whether these factors are associated with KT outcome (ie, with or without transplant), type of KT received (ie, living donor or deceased donor), and time to KT. Future studies should also assess whether other patient factors (eg, KT knowledge) change after exposure to a streamlined care model or should use qualitative methods to explore patients’ experiences as they progress through a concierge-based clinic approach.

## Notes

Supplement 1. **eTable 1.** Description of Study Variables **eTable 2.** Missingness of Outcome Variables at Baseline and Follow-Up by Waitlisting Status at Follow-Up **eTable 3.** Correlation Matrix for Baseline Perception of Health Care Variables **eTable 4.** Least-Squares Mean of Perceptions of Health Care Scores by Race and Time **eTable 5.** Complete Output for Multivariable Logistic Regression Examining Main Effects, Experiences of Discrimination in Health Care **eTable 6.** Complete Output for Multivariable Linear Regression Examining Main Effects, Perceived Racism in Health Care **eTable 7.** Complete Output for Multivariable Linear Regression Examining Main Effects, Medical Mistrust **eTable 8.** Complete Output for Multivariable Linear Regression Examining Main Effects, Trust in Physician **eTable 9.** Complete Output for Multivariable Logistic Regression Examining Race-by-Time, Experiences of Discrimination in Health Care **eTable 10.** Complete Output for Multivariable Regression Examining Race-by-Time, Perceived Racism in Health Care **eTable 11.** Complete Output for Multivariable Regression Examining Race by Time, Medical Mistrust **eTable 12.** Complete Output for Multivariable Regression Examining Race by Time, Trust in Physician
eReferences

Supplement 2.
Data Sharing Statement
