# Supplementary material for: Health Care Perceptions and a Concierge-Based Transplant Evaluation for Patients With Kidney Disease
Source: JAMA Netw Open. 2024 Nov 26;7(11):e2447335. doi: 10.1001/jamanetworkopen.2024.47335 (PMC11600232; doi:10.1001/jamanetworkopen.2024.47335)
Supplement: Supplement 1. — eTable 1. Description of Study Variables eTable 2. Missingness of Outcome Variables at Baseline and Follow-Up by Waitlisting Status at Follow-Up eTable 3. Correlation Matrix for Baseline Perception of Health Care Variables eTable 4. Least-Squares Mean of Perceptions of Health Care Scores by Race and Time eTable 5. Complete Output for Multivariable Logistic Regression Examining Main Effects, Experiences of Discrimination in Health Care eTable 6. Complete Output for Multivariable Linear Regression Examining Main Effects, Perceived Racism in Health Care eTable 7. Complete Output for Multivariable Linear Regression Examining Main Effects, Medical Mistrust eTable 8. Complete Output for Multivariable Linear Regression Examining Main Effects, Trust in Physician eTable 9. Complete Output for Multivariable Logistic Regression Examining Race-by-Time, Experiences of Discrimination in Health Care eTable 10. Complete Output for Multivariable Regression Examining Race-by-Time, Perceived Racism in Health Care eTable 11. Complete Output for Multivariable Regression Examining Race by Time, Medical Mistrust eTable 12. Complete Output for Multivariable Regression Examining Race by Time, Trust in Physician eReferences [file jamanetwopen-e2447335-s001.pdf]

Supplemental Online Content

Vélez-Bermúdez M, Leyva Y, Loor JM, et al. Health care perceptions and a concierge-based transplant evaluation for patients with kidney disease. *JAMA Netw Open*. 2024; 7(11):e2447335. doi:10.1001/jamanetworkopen.2024.47335

eTable 1. Description of Study Variables
eTable 2. Missingness of Outcome Variables at Baseline and Follow-Up by Waitlisting Status at Follow-Up
eTable 3. Correlation Matrix for Baseline Perception of Health Care Variables
eTable 4. Least-Squares Mean of Perceptions of Health Care Scores by Race and Time
eTable 5. Complete Output for Multivariable Logistic Regression Examining Main Effects, Experiences of Discrimination in Health Care
eTable 6. Complete Output for Multivariable Linear Regression Examining Main Effects, Perceived Racism in Health Care
eTable 7. Complete Output for Multivariable Linear Regression Examining Main Effects, Medical Mistrust
eTable 8. Complete Output for Multivariable Linear Regression Examining Main Effects, Trust in Physician
eTable 9. Complete Output for Multivariable Logistic Regression Examining Race-by-Time, Experiences of Discrimination in Health Care
eTable 10. Complete Output for Multivariable Regression Examining Race-by-Time, Perceived Racism in Health Care
eTable 11. Complete Output for Multivariable Regression Examining Race by Time, Medical Mistrust
eTable 12. Complete Output for Multivariable Regression Examining Race by Time, Trust in Physician
eReferences

This supplemental material has been provided by the authors to give readers additional information about their work.

© 2024 Vélez-Bermúdez M. *JAMA Network Open*.

eTable 1. Description of Study Variables

Study variables	Description and scoring	Cronbach's α[1]
Socio-demographics		
Race	Patient-reported and dichotomized as "White, non-Hispanic" or "Black, non-Hispanic"	Not applicable
Age	Patient-reported; years	Not applicable
Sex	Patient-reported; male or female	Not applicable
Education	Patient-reported and dichotomized as "12th grade or less" or "Some education beyond 12th grade".	Not applicable
Employment status	Patient-reported and dichotomized as "Employed part-time or full-time" or "Unemployed, retired, or on disability"	Not applicable
Family income	Patient-reported and dichotomized as "Less than $50,000 per year" or "$50,000 or greater per year"	Not applicable
Health Insurance	Obtained from the electronic medical record and classified as private insurance only, public insurance only, or both private and public.	Not applicable
Marital status	Patient-reported and dichotomized as "Single, separated or divorced, widowed", or "Married or in a domestic partnership."	Not applicable
Number of people in one's social network (i.e., potential living kidney donors)	Patient-reported number of the network of potential living donors available for evaluation was determined by asking participants to indicate how many living relatives and friends they had aged 18–70 years of age. In the present sample, the number of potential living donors available to the participant ranged from 0 – 150.	Not applicable
Transplant factors		
Transplant knowledge [1]	Participants were assessed on their knowledge about transplant using a 19-item KT Knowledge Survey. Items 1 – 8 were multiple choice, and items 9 – 19 were "True" or "False." Each item answered correctly adds 1 to total score. Scores in the present sample ranged from 0 – 18.	0.87
Number of learning activities [2]	Participants reported the type and number of KT-related learning activities (e.g., reading brochures, online research) with greater numbers indicating engagement in more learning activities. Scores in the present sample ranged from 0 – 4.	0.50
Hours engaged in learning activities [2]	Participants reported the amount of time spent in KT-related learning activities (e.g., reading brochures, online research). In the present sample, we categorized responses to 1 (0 – 2 hours of learning), 2 (greater than 2 hours, less than or equal to 5 hours of learning), or 3 (greater than 5 hours of learning).	0.57

© 2024 Vélez-Bermúdez M. *JAMA Network Open*.

Study variables	Description and scoring	Cronbach's α[1]
Total transplant Concerns [2,3]	In a 24-item assessment, participants reported which of 24 common transplant-related concerns were most important in influencing their decision to pursue transplant. Item responses ranged from 1 ("Not important") to 5 ("Extremely important"), and higher scores reflect greater concern. In the present sample, scores ranged from 16 – 60.	0.82
Medical factors (abstracted from EMR review)		
Body Mass Index (BMI)	Calculated with patient height and weight (obtained from medical record) using NHLBI's calculator: https://www.nhlbi.nih.gov/health/educational/lose_wt/BMI/bmicalc.htm	Not applicable
Charlson co-morbidity Index [4]	The score was obtained using patient data obtained from the medical record reflecting the number and severity of co-morbid health conditions. The score is continuous with a possible range of 0 to 33 with higher scores reflecting a more significant type of comorbidity. In the present sample, scores ranged from 2 – 13.	Not applicable
Dialysis	Determined from the either the medical record or self-report (either Hemodialysis, Peritoneal Dialysis, or not on dialysis).	Not applicable
Dialysis duration (in years)	Determined from medical records. In the present sample, years on dialysis ranged from 0 – 15.41. In the present sample, we categorized responses to 1 (never on dialysis), 2 (less than 1 year on dialysis), 3 (1 – 5 years on dialysis), or 4 (5 or more years on dialysis).	Not applicable
Waitlisting status at Follow-up	Determined from medical records at time of follow-up. The following labels were assigned to each participant:<br>-Accepted for KT waitlisting<br>-Rejected for KT waitlisting (i.e., closed for medical issues)<br>-Evaluation closed or incomplete	Not applicable
Days to index evaluation completion.	Obtained from medical record. Number of days between the attended initial clinic KT evaluation appointment and the date of initial evaluation completion.	Not applicable
Psychosocial and cultural factors		
Family loyalty [5]	A sixteen-item measure assessing participants' beliefs on family loyalty and mutual support. Responses range from 1 ("Strongly Disagree") to 5 ("Strongly Agree"). The measure was scored by taking the mean score of each item. Higher scores reflect greater family loyalty. In this sample, family loyalty scores ranged from 1.44 – 5.	0.84
Health literacy [5]	A three-item measure assessing participants' understanding about their own health information, with scores ranging from 1 ("Always," "Extremely") to	0.68

© 2024 Vélez-Bermúdez M. *JAMA Network Open*.

Study variables	Description and scoring	Cronbach's α[1]
	5 ("Never," "Not at all"). Higher scores reflect higher literacy. The measure was scored by taken the mean score of each item. In this sample, health literacy scores ranged from 1 – 5.	
Any religious objection to LDKT [6]	Adapted subscale of the 8-item Organ Donation Attitude Survey (ODAS). From these questions, we dichotomized responses into either "Any objection" v. "No objection" to transplant.	0.66
Social support [7]	A twelve-item measure from the Interpersonal Support Evaluation List (ISEL-12) assessing participants' perceived availability of 3 separate functions of social support: "tangible," "appraisal," and "belonging". Scores ranged from 1 ("Definitely false") to 4 ("Definitely true"). Higher scores reflect greater social support. Sum scores were taken from participant responses, and scores ranged from 12 – 48.	0.86
Anxiety [8]	6-item Brief Symptom Inventory (BSI) Anxiety subscale where participants report the extent to which they indicate how bothered or distressed they have felt in the past 2 weeks by several symptoms. Responses ranged from 1 ("Not at all") to 5 ("Extremely"). Responses were dichotomized to reflect "No anxiety" or "Moderate to severe anxiety."	0.86
Depression [8]	6-item Brief Symptom Inventory (BSI) Depression subscale where participants report the extent to which they indicate how bothered or distressed they have felt in the past 2 weeks by several symptoms. Responses ranged from 1 ("Not at all") to 5 ("Extremely"). Responses were dichotomized to reflect "No depression" or "Moderate to severe depression."	0.85

Outcome variables	Description and scoring	Cronbach's α[1]
Experiences of discrimination in healthcare [9,10]	A seven-item measure assessing participants' personal experiences of race/ethnicity-based discrimination during interactions with healthcare providers (sample item: "When getting health care, have you ever had any of the following things happen to you because of your Race or Ethnicity? Received poorer service than others because of your race and ethnicity"). Responses range from 1 ("Never") to 5 ("Always"). For this sample, responses were dichotomized to "Ever experienced discrimination" to "Never experienced discrimination."	0.89
Perceived racism in healthcare [11,12]	A four-item measure of patient beliefs of the extent to which racism is common in healthcare (sample item: "Racial discrimination in a doctor's office is common"). Responses range from 1 ("Strongly Disagree") to 5 ("Strongly Agree"). Higher scores	0.75

© 2024 Vélez-Bermúdez M. *JAMA Network Open*.

Study variables	Description and scoring	Cronbach's α[1]
	reflect higher perception of racism. The measure was scored by taking the mean score of each item. In this sample, perceived racism in healthcare scores ranged from 1 – 5.	
Medical mistrust [13]	A seven-item measure assessing participant beliefs that healthcare organizations and hospital systems are untrustworthy, incompetent, and not acting in patients' best interests (sample item: "Patient have sometimes been deceived or misled by healthcare organizations.") Responses range from 1 ("Strongly Disagree") to 5 ("Strongly Agree"). Higher scores reflect higher medical mistrust. The measure was scored by taking the mean score of each item. In this sample, medical mistrust scores ranged from 1 – 5.	0.78
Trust in physician [14]	An eleven-item measure assessing the extent to which participants trust their physicians (sample item: "If my doctor tells me something is so, then it must be true"). Responses range from 1 ("Totally Disagree") to 5 ("Totally Agree"). Higher scores reflect higher trust in physician. The measure was scored by taking the mean score of each item. In this sample, trust in physician scores ranged from 1.45 – 5.	0.85

[1] Cronbach's alpha values calculated for total scales within the current study sample

© 2024 Vélez-Bermúdez M. *JAMA Network Open*.

eTable 2. Missingness of Outcome Variables at Baseline and Follow-Up by Waitlisting Status at Follow-Up

	Accepted for transplant (n = 462)	Rejected for transplant (n = 138)	Evaluation closed or incomplete (n = 220)
Experiences of Discrimination, n (column %):			
Baseline	0 (0.0)	0 (0.0)	0 (0.0)
Follow-up	2 (0.4)	0 (0.0)	0 (0.0)
Perceived Racism, n (column %):			
Baseline	3 (0.6)	1 (0.7)	0 (0.0)
Follow-up	11 (2.4)	3 (2.2)	1 (0.5)
Medical Mistrust, n (column %):			
Baseline	0 (0.0)	2 (1.4)	0 (0.0)
Follow-up	8 (1.7)	3 (2.2)	2 (0.9)
Trust in Physician, n (column %):			
Baseline	0 (0.0)	0 (0.0)	0 (0.0)
Follow-up	3 (0.6)	3 (2.2)	1 (0.5)

© 2024 Vélez-Bermúdez M. *JAMA Network Open*.

eTable 3. Correlation Matrix for Baseline Perception of Health Care Variables

	Experiences of Discrimination	Perceived Racism	Medical Mistrust	Trust in Physician
Experiences of Discrimination	-	-	-	-
Perceived Racism	0.46 **	-	-	-
Medical Mistrust	0.33 **	0.42 **	-	-
Trust in Physician	-0.23 **	-0.21 **	-0.35 **	-

Note: Pearson correlation reported for all. ***p* < .001

© 2024 Vélez-Bermúdez M. *JAMA Network Open*.

eTable 4. Least-Squares Mean of Perceptions of Health Care Scores by Race and Time

	Experiences of Discrimination	Perceived Racism	Medical Mistrust	Trust in Physician
	OR (95% CI)	β (SE)	β (SE)	β (SE)
Black participants at Baseline	1.64 (0.83, 3.22)	2.89 (0.09)	3.53 (0.09)	3.85 (0.07)
White participants at Baseline	0.16 (0.08, 0.30)	2.32 (0.08)	3.11 (0.08)	3.89 (0.06)
Black participants at Follow-up	0.44 (0.22, 0.87)	2.78 (0.09)	3.36 (0.09)	3.71 (0.07)
White participants at Follow-up	0.06 (0.03, 0.12)	2.26 (0.08)	3.02 (0.08)	3.92 (0.06)

Note: Values are the linear combination of the estimated effects from each model testing the Race-by-Time interaction. These same values are reflected in Figure 2 of the manuscript.

© 2024 Vélez-Bermúdez M. *JAMA Network Open*.

eTable 5. Complete Output for Multivariable Logistic Regression Examining Main Effects, Experiences of Discrimination in Health Care

Parameter		Estimate	SE	t-value	Pr > |Z|
**Intercept**		2.1848	1.215	1.8	0.0725
**Race (Ref: White)**	Black	2.1906	0.2381	9.2	<.0001
**Time (Ref: Baseline)**	Follow-up	-1.1126	0.1652	-6.73	<.0001
**Age**		-0.00265	0.008088	-0.33	0.7435
**Gender (Ref: Male)**	Female	-0.1035	0.1897	-0.55	0.5856
**Marital status (Ref: Single)**	In a marital or domestic partnership	-0.00217	0.2076	-0.01	0.9917
**Family income (Ref: less than $50K)**	$50K or Greater	-0.02384	0.2482	-0.1	0.9235
**Employment status (Ref: Unemployed)**	Part- or full-time	-0.3365	0.2428	-1.39	0.1661
**Transplant knowledge**		-0.07432	0.0353	-2.11	0.0356
**Number of learning activities**		-0.02354	0.1154	-0.2	0.8385
**Hours of learning activities (Ref: 0 – 2 hours)**	Greater than 2, less than or equal to 5 hours	0.691	0.2687	2.57	0.0103
**Hours of learning activities (Ref: 0 – 2 hours)**	Greater than 5 hours	0.5304	0.2862	1.85	0.0642
**Transplant concerns**		0.008478	0.01229	0.69	0.4904
**Insurance status (Ref: Private only)**	Both Private and Public	-0.1338	0.289	-0.46	0.6436
**Insurance status (Ref: Private only)**	Public only	-0.2962	0.3055	-0.97	0.3327
**Days from evaluation to Follow-up**		-0.00026	0.000622	-0.42	0.6748
**Waitlist status at Follow-up (Ref: Accepted for transplant)**	Evaluation closed or incomplete	0.4727	0.235	2.01	0.0446
**Waitlist status at Follow-up (Ref: Accepted for transplant)**	Rejected for transplant	0.1008	0.2543	0.4	0.692
**Dialysis duration categorized (Ref: no dialysis)**	Greater than 0, less than 1 year	-1.7657	0.7013	-2.52	0.012
**Dialysis duration categorized (Ref: no dialysis)**	Greater than or equal to 1 year, less than 5 years	-1.6013	0.7198	-2.22	0.0264
**Dialysis duration categorized (Ref: no dialysis)**	Greater than or equal to 5 years	-1.0756	0.7885	-1.36	0.1729
**Dialysis modality (Ref: Not on dialysis)**	Hemodialysis	1.4343	0.6907	2.08	0.0382
**Dialysis modality (Ref: Not on dialysis)**	Peritoneal dialysis	1.136	0.7372	1.54	0.1237

© 2024 Vélez-Bermúdez M. *JAMA Network Open*.

Parameter		Estimate	SE	t-value	Pr > |Z|
**Social support**		-0.05789	0.01519	-3.81	0.0001
**Anxiety (Ref: No anxiety)**	Moderate anxiety or greater	-0.5606	0.4966	-1.13	0.2593
**Depression (Ref: No depression)**	Moderate depression or greater	0.9395	0.4302	2.18	0.0293
**Network of potential living kidney donors (square root transformation)**		0.02568	0.05441	0.47	0.6371
**Health literacy**		-0.235	0.0957	-2.46	0.0143
**Family loyalty**		-0.0332	0.1676	-0.2	0.843
**Religious objection (Ref: No religious objection to transplant)**	Any religious objection to transplant	-0.2901	0.186	-1.56	0.1193

© 2024 Vélez-Bermúdez M. *JAMA Network Open*.

eTable 6. Complete Output for Multivariable Linear Regression Examining Main Effects, Perceived Racism in Health Care

**Effect**		**Estimate**	**Standard Error**	**t Value**	**Pr > |t|**
**Intercept**		2.76	0.3222	8.56	<.0001
**Race (Ref: White)**	Black	0.5502	0.05967	9.22	<.0001
**Time (Ref: Baseline)**	Follow-up	-0.07107	0.02737	-2.6	0.0096
**Age**		-0.0003	0.002155	-0.14	0.8884
**Gender (Ref: Male)**	Female	0.09058	0.05006	1.81	0.0708
**Marital status (Ref: Single)**	In a marital or domestic partnership	-0.03532	0.0551	-0.64	0.5217
**Family income (Ref: less than $50K)**	$50K or Greater	0.09583	0.06364	1.51	0.1325
**Employment status (Ref: Unemployed)**	Part- or full-time	0.004811	0.06085	0.08	0.937
**Transplant knowledge**		-0.00887	0.00945	-0.94	0.3481
**Number of learning activities**		0.03865	0.03009	1.28	0.1994
**Hours of learning activities (Ref: 0 – 2 hours)**	Greater than 2, less than or equal to 5 hours	0.003519	0.07062	0.05	0.9603
**Hours of learning activities (Ref: 0 – 2 hours)**	Greater than 5 hours	0.02718	0.07469	0.36	0.716
**Transplant concerns**		0.001269	0.003234	0.39	0.6949
**Insurance status (Ref: Private only)**	Both Private and Public	0.03031	0.07402	0.41	0.6823
**Insurance status (Ref: Private only)**	Public only	-0.04202	0.07973	-0.53	0.5983
**Days from evaluation to Follow-up**		0.000168	0.000167	1	0.3157
**Waitlist status at Follow-up (Ref: Accepted for transplant)**	Evaluation closed or incomplete	-0.01145	0.06363	-0.18	0.8572
**Waitlist status at Follow-up (Ref: Accepted for transplant)**	Rejected for transplant	0.01745	0.06774	0.26	0.7968
**Dialysis duration categorized (Ref: no dialysis)**	Greater than 0, less than 1 year	-0.09297	0.1551	-0.6	0.549
**Dialysis duration categorized (Ref: no dialysis)**	Greater than or equal to 1 year, less than 5 years	-0.1697	0.1623	-1.05	0.2961
**Dialysis duration categorized (Ref: no dialysis)**	Greater than or equal to 5 years	-0.1025	0.1868	-0.55	0.5831
**Dialysis modality (Ref: Not on dialysis)**	Hemodialysis	0.09266	0.1529	0.61	0.5446
**Dialysis modality (Ref: Not on dialysis)**	Peritoneal dialysis	0.02509	0.1657	0.15	0.8797
**Social support**		-0.00972	0.004107	-2.37	0.0182

© 2024 Vélez-Bermúdez M. *JAMA Network Open*.

Effect		Estimate	Standard Error	t Value	Pr > |t|
**Anxiety (Ref: No anxiety)**	Moderate anxiety or greater	-0.06841	0.13	-0.53	0.599
**Depression (Ref: No depression)**	Moderate depression or greater	0.3603	0.124	2.9	0.0038
**Network of potential living kidney donors (square root transformation)**		0.0122	0.01445	0.84	0.399
**Health literacy**		-0.01685	0.02541	-0.66	0.5075
**Family loyalty**		-0.07883	0.04496	-1.75	0.0799
**Religious objection (Ref: No religious objection to transplant)**	Any religious objection to transplant	-0.02468	0.04886	-0.51	0.6136

© 2024 Vélez-Bermúdez M. *JAMA Network Open*.

eTable 7. Complete Output for Multivariable Linear Regression Examining Main Effects, Medical Mistrust

Effect		Estimate	Standard Error	t Value	Pr > |t|
**Intercept**		3.3029	0.3028	10.91	<.0001
**Race (Ref: White)**	Black	0.3783	0.05618	6.73	<.0001
**Time (Ref: Baseline)**	Follow-up	-0.1102	0.02413	-4.57	<.0001
**Age**		-0.00184	0.002027	-0.91	0.3643
**Gender (Ref: Male)**	Female	0.06344	0.04709	1.35	0.1784
**Marital status (Ref: Single)**	In a marital or domestic partnership	0.04237	0.05186	0.82	0.4142
**Family income (Ref: less than $50K)**	$50K or Greater	-0.06128	0.05976	-1.03	0.3055
**Employment status (Ref: Unemployed)**	Part- or full-time	-0.08347	0.0572	-1.46	0.1449
**Transplant knowledge**		-0.01669	0.008876	-1.88	0.0604
**Number of learning activities**		0.03917	0.02832	1.38	0.1669
**Hours of learning activities (Ref: 0 – 2 hours)**	Greater than 2, less than or equal to 5 hours	0.02329	0.06651	0.35	0.7263
**Hours of learning activities (Ref: 0 – 2 hours)**	Greater than 5 hours	-0.00518	0.07032	-0.07	0.9413
**Transplant concerns**		0.008736	0.003042	2.87	0.0042
**Insurance status (Ref: Private only)**	Both Private and Public	0.04085	0.06953	0.59	0.557
**Insurance status (Ref: Private only)**	Public only	-0.131	0.07496	-1.75	0.081
**Days from evaluation to Follow-up**		0.0003	0.000157	1.91	0.057
**Waitlist status at Follow-up (Ref: Accepted for transplant)**	Evaluation closed or incomplete	0.02961	0.05991	0.49	0.6213
**Waitlist status at Follow-up (Ref: Accepted for transplant)**	Rejected for transplant	0.09406	0.06378	1.47	0.1407
**Dialysis duration categorized (Ref: no dialysis)**	Greater than 0, less than 1 year	-0.05876	0.146	-0.4	0.6874
**Dialysis duration categorized (Ref: no dialysis)**	Greater than or equal to 1 year, less than 5 years	-0.06892	0.1528	-0.45	0.652
**Dialysis duration categorized (Ref: no dialysis)**	Greater than or equal to 5 years	-0.07094	0.1756	-0.4	0.6864
**Dialysis modality (Ref: Not on dialysis)**	Hemodialysis	0.08563	0.1439	0.6	0.552
**Dialysis modality (Ref: Not on dialysis)**	Peritoneal dialysis	0.05809	0.156	0.37	0.7097
**Social support**		-0.01452	0.003858	-3.76	0.0002

© 2024 Vélez-Bermúdez M. *JAMA Network Open*.

Effect		Estimate	Standard Error	t Value	Pr > |t|
**Anxiety (Ref: No anxiety)**	Moderate anxiety or greater	0.313	0.1224	2.56	0.0108
**Depression (Ref: No depression)**	Moderate depression or greater	0.1793	0.1167	1.54	0.125
**Network of potential living kidney donors (square root transformation)**		0.0071	0.01359	0.52	0.6015
**Health literacy**		-0.02832	0.02385	-1.19	0.2354
**Family loyalty**		-0.001	0.04228	-0.02	0.9811
**Religious objection (Ref: No religious objection to transplant)**	Any religious objection to transplant	0.04648	0.04598	1.01	0.3124

© 2024 Vélez-Bermúdez M. *JAMA Network Open*.

eTable 8. Complete Output for Multivariable Linear Regression Examining Main Effects, Trust in Physician

Effect		Estimate	Standard Error	t Value	Pr > |t|
**Intercept**		2.9301	0.2451	11.96	<.0001
**Race (Ref: White)**	Black	-0.1301	0.0455	-2.86	0.0044
**Time (Ref: Baseline)**	Follow-up	-0.01469	0.02332	-0.63	0.529
**Age**		-0.00016	0.001643	-0.1	0.924
**Gender (Ref: Male)**	Female	-0.0389	0.03813	-1.02	0.308
**Marital status (Ref: Single)**	In a marital or domestic partnership	-0.04678	0.042	-1.11	0.2657
**Family income (Ref: less than $50K)**	$50K or Greater	0.06132	0.04841	1.27	0.2056
**Employment status (Ref: Unemployed)**	Part- or full-time	0.03018	0.04628	0.65	0.5146
**Transplant knowledge**		0.00798	0.007177	1.11	0.2665
**Number of learning activities**		-0.00895	0.02293	-0.39	0.6964
**Hours of learning activities (Ref: 0 – 2 hours)**	Greater than 2, less than or equal to 5 hours	-0.02139	0.05384	-0.4	0.6913
**Hours of learning activities (Ref: 0 – 2 hours)**	Greater than 5 hours	0.00563	0.05694	0.1	0.9213
**Transplant concerns**		0.001436	0.002462	0.58	0.56
**Insurance status (Ref: Private only)**	Both Private and Public	-0.00481	0.05631	-0.09	0.932
**Insurance status (Ref: Private only)**	Public only	0.0445	0.06072	0.73	0.4639
**Days from evaluation to Follow-up**		-0.00019	0.000127	-1.46	0.1456
**Waitlist status at Follow-up (Ref: Accepted for transplant)**	Evaluation closed or incomplete	-0.03207	0.04852	-0.66	0.5089
**Waitlist status at Follow-up (Ref: Accepted for transplant)**	Rejected for transplant	-0.04593	0.05164	-0.89	0.3741
**Dialysis duration categorized (Ref: no dialysis)**	Greater than 0, less than 1 year	0.1719	0.1183	1.45	0.1466
**Dialysis duration categorized (Ref: no dialysis)**	Greater than or equal to 1 year, less than 5 years	0.1812	0.1238	1.46	0.1436
**Dialysis duration categorized (Ref: no dialysis)**	Greater than or equal to 5 years	0.05649	0.1424	0.4	0.6916
**Dialysis modality (Ref: Not on dialysis)**	Hemodialysis	-0.218	0.1166	-1.87	0.0619
**Dialysis modality (Ref: Not on dialysis)**	Peritoneal dialysis	-0.204	0.1264	-1.61	0.107

© 2024 Vélez-Bermúdez M. *JAMA Network Open*.

Effect		Estimate	Standard Error	t Value	Pr > |t|
**Social support**		0.01861	0.003125	5.95	<.0001
**Anxiety (Ref: No anxiety)**	Moderate anxiety or greater	-0.01099	0.0992	-0.11	0.9118
**Depression (Ref: No depression)**	Moderate depression or greater	-0.2243	0.0946	-2.37	0.018
**Network of potential living kidney donors (square root transformation)**		-0.00114	0.011	-0.1	0.9174
**Health literacy**		0.03488	0.01932	1.81	0.0713
**Family loyalty**		0.06506	0.03418	1.9	0.0573
**Religious objection (Ref: No religious objection to transplant)**	Any religious objection to transplant	-0.08105	0.03722	-2.18	0.0297

© 2024 Vélez-Bermúdez M. *JAMA Network Open*.

eTable 9. Complete Output for Multivariable Logistic Regression Examining Race-by-Time, Experiences of Discrimination in Health Care

Parameter		Estimate	SE	t-value	Pr > |Z|
**Intercept**		2.1655	1.228	1.76	0.0782
**Race (Ref: White)**	Black	2.3582	0.2855	8.26	<.0001
**Time (Ref: Baseline)**	Follow-up	-0.9923	0.2048	-4.84	<.0001
**Race * Time**	Black * Follow-up	-0.3195	0.3178	-1.01	0.315
**Age**		-0.00242	0.008176	-0.3	0.7675
**Gender (Ref: Male)**	Female	-0.118	0.1917	-0.62	0.5384
**Marital status (Ref: Single)**	In a marital or domestic partnership	0.007948	0.2095	0.04	0.9698
**Family income (Ref: less than $50K)**	$50K or Greater	-0.0226	0.2504	-0.09	0.9281
**Employment status (Ref: Unemployed)**	Part- or full-time	-0.3199	0.2448	-1.31	0.1917
**Transplant knowledge**		-0.07495	0.03568	-2.1	0.036
**Number of learning activities**		-0.01577	0.1166	-0.14	0.8925
**Hours of learning activities (Ref: 0 – 2 hours)**	Greater than 2, less than or equal to 5 hours	0.6811	0.2711	2.51	0.0122
**Hours of learning activities (Ref: 0 – 2 hours)**	Greater than 5 hours	0.5244	0.2889	1.82	0.0699
**Transplant concerns**		0.008677	0.01242	0.7	0.4849
**Insurance status (Ref: Private only)**	Both Private and Public	-0.131	0.2916	-0.45	0.6534
**Insurance status (Ref: Private only)**	Public only	-0.2927	0.3084	-0.95	0.3428
**Days from evaluation to Follow-up**		-0.00024	0.000628	-0.38	0.7066
**Waitlist status at Follow-up (Ref: Accepted for transplant)**	Evaluation closed or incomplete	0.4535	0.2372	1.91	0.0563
**Waitlist status at Follow-up (Ref: Accepted for transplant)**	Rejected for transplant	0.09219	0.2566	0.36	0.7195
**Dialysis duration categorized (Ref: no dialysis)**	Greater than 0, less than 1 year	-1.7654	0.708	-2.49	0.0129
**Dialysis duration categorized (Ref: no dialysis)**	Greater than or equal to 1 year, less than 5 years	-1.5874	0.7266	-2.18	0.0292
**Dialysis duration categorized (Ref: no dialysis)**	Greater than or equal to 5 years	-1.077	0.7968	-1.35	0.1769
**Dialysis modality (Ref: Not on dialysis)**	Hemodialysis	1.4421	0.6976	2.07	0.039
**Dialysis modality (Ref: Not on dialysis)**	Peritoneal dialysis	1.1511	0.7444	1.55	0.1224
**Social support**		-0.05902	0.01537	-3.84	0.0001

© 2024 Vélez-Bermúdez M. *JAMA Network Open*.

Parameter		Estimate	SE	t-value	Pr > |Z|
**Anxiety (Ref: No anxiety)**	Moderate anxiety or greater	-0.5643	0.501	-1.13	0.2603
**Depression (Ref: No depression)**	Moderate depression or greater	0.9276	0.434	2.14	0.0329
**Network of potential living kidney donors (square root transformation)**		0.02551	0.05496	0.46	0.6426
**Health literacy**		-0.2423	0.09679	-2.5	0.0125
**Family loyalty**		-0.03253	0.1693	-0.19	0.8477
**Religious objection (Ref: No religious objection to transplant)**	Any religious objection to transplant	-0.3087	0.188	-1.64	0.1011

© 2024 Vélez-Bermúdez M. *JAMA Network Open*.

eTable 10. Complete Output for Multivariable Regression Examining Race-by-Time, Perceived Racism in Health Care

Effect		Estimate	Standard Error	t Value	Pr > |t|
**Intercept**		2.754	0.3223	8.54	<.0001
**Race (Ref: White)**	Black	0.5779	0.06742	8.57	<.0001
**Time (Ref: Baseline)**	Follow-up	-0.05709	0.03161	-1.81	0.0713
**Race * Time**	Black * Follow-up	-0.05591	0.06321	-0.88	0.3767
**Age**		-0.00031	0.002155	-0.14	0.8867
**Gender (Ref: Male)**	Female	0.09049	0.05006	1.81	0.0711
**Marital status (Ref: Single)**	In a marital or domestic partnership	-0.03527	0.0551	-0.64	0.5223
**Family income (Ref: less than $50K)**	$50K or Greater	0.09593	0.06364	1.51	0.1321
**Employment status (Ref: Unemployed)**	Part- or full-time	0.004803	0.06085	0.08	0.9371
**Transplant knowledge**		-0.00889	0.00945	-0.94	0.3469
**Number of learning activities**		0.03873	0.03009	1.29	0.1985
**Hours of learning activities (Ref: 0 – 2 hours)**	Greater than 2, less than or equal to 5 hours	0.003252	0.07062	0.05	0.9633
**Hours of learning activities (Ref: 0 – 2 hours)**	Greater than 5 hours	0.02705	0.07469	0.36	0.7173
**Transplant concerns**		0.001265	0.003234	0.39	0.6957
**Insurance status (Ref: Private only)**	Both Private and Public	0.03034	0.07402	0.41	0.682
**Insurance status (Ref: Private only)**	Public only	-0.04213	0.07973	-0.53	0.5974
**Days from evaluation to Follow-up**		0.000167	0.000167	1	0.3176
**Waitlist status at Follow-up (Ref: Accepted for transplant)**	Evaluation closed or incomplete	-0.01142	0.06363	-0.18	0.8576
**Waitlist status at Follow-up (Ref: Accepted for transplant)**	Rejected for transplant	0.01732	0.06774	0.26	0.7983
**Dialysis duration categorized (Ref: no dialysis)**	Greater than 0, less than 1 year	-0.09291	0.1551	-0.6	0.5493
**Dialysis duration categorized (Ref: no dialysis)**	Greater than or equal to 1 year, less than 5 years	-0.1698	0.1623	-1.05	0.296
**Dialysis duration categorized (Ref: no dialysis)**	Greater than or equal to 5 years	-0.1019	0.1868	-0.55	0.5856
**Dialysis modality (Ref: Not on dialysis)**	Hemodialysis	0.09281	0.1529	0.61	0.544
**Dialysis modality (Ref: Not on dialysis)**	Peritoneal dialysis	0.02518	0.1657	0.15	0.8793
**Social support**		-0.00973	0.004107	-2.37	0.0181

© 2024 Vélez-Bermúdez M. *JAMA Network Open*.

Effect		Estimate	Standard Error	t Value	Pr > |t|
**Anxiety (Ref: No anxiety)**	Moderate anxiety or greater	-0.06844	0.13	-0.53	0.5988
**Depression (Ref: No depression)**	Moderate depression or greater	0.3603	0.124	2.9	0.0038
**Network of potential living kidney donors (square root transformation)**		0.01218	0.01445	0.84	0.3997
**Health literacy**		-0.0168	0.02541	-0.66	0.5088
**Family loyalty**		-0.07878	0.04496	-1.75	0.0801
**Religious objection (Ref: No religious objection to transplant)**	Any religious objection to transplant	-0.02465	0.04886	-0.5	0.6141

© 2024 Vélez-Bermúdez M. *JAMA Network Open*.

eTable 11. Complete Output for Multivariable Regression Examining Race by Time, Medical Mistrust

Effect		Estimate	Standard Error	t Value	Pr > |t|
**Intercept**		3.2946	0.3029	10.88	<.0001
**Race (Ref: White)**	Black	0.412	0.06265	6.58	<.0001
**Time (Ref: Baseline)**	Follow-up	-0.09329	0.02786	-3.35	9E-04
**Race * Time**	Black * Follow-up	-0.06758	0.05568	-1.21	0.225
**Age**		-0.00184	0.002027	-0.91	0.364
**Gender (Ref: Male)**	Female	0.06331	0.04709	1.34	0.179
**Marital status (Ref: Single)**	In a marital or domestic partnership	0.04243	0.05186	0.82	0.414
**Family income (Ref: less than $50K)**	$50K or Greater	-0.06148	0.05976	-1.03	0.304
**Employment status (Ref: Unemployed)**	Part- or full-time	-0.08324	0.0572	-1.46	0.146
**Transplant knowledge**		-0.01673	0.008876	-1.88	0.06
**Number of learning activities**		0.03919	0.02832	1.38	0.167
**Hours of learning activities (Ref: 0 – 2 hours)**	Greater than 2, less than or equal to 5 hours	0.02321	0.06651	0.35	0.727
**Hours of learning activities (Ref: 0 – 2 hours)**	Greater than 5 hours	-0.00524	0.07032	-0.07	0.941
**Transplant concerns**		0.008737	0.003042	2.87	0.004
**Insurance status (Ref: Private only)**	Both Private and Public	0.0409	0.06953	0.59	0.557
**Insurance status (Ref: Private only)**	Public only	-0.1312	0.07496	-1.75	0.081
**Days from evaluation to Follow-up**		0.000299	0.000157	1.91	0.057
**Waitlist status at Follow-up (Ref: Accepted for transplant)**	Evaluation closed or incomplete	0.0296	0.05991	0.49	0.621
**Waitlist status at Follow-up (Ref: Accepted for transplant)**	Rejected for transplant	0.09405	0.06378	1.47	0.141
**Dialysis duration categorized (Ref: no dialysis)**	Greater than 0, less than 1 year	-0.0587	0.146	-0.4	0.688
**Dialysis duration categorized (Ref: no dialysis)**	Greater than or equal to 1 year, less than 5 years	-0.06907	0.1528	-0.45	0.651
**Dialysis duration categorized (Ref: no dialysis)**	Greater than or equal to 5 years	-0.07085	0.1756	-0.4	0.687
**Dialysis modality (Ref: Not on dialysis)**	Hemodialysis	0.0858	0.1439	0.6	0.551
**Dialysis modality (Ref: Not on dialysis)**	Peritoneal dialysis	0.05819	0.156	0.37	0.709

© 2024 Vélez-Bermúdez M. *JAMA Network Open*.

Effect		Estimate	Standard Error	t Value	Pr > |t|
**Social support**		-0.01453	0.003858	-3.77	2E-04
**Anxiety (Ref: No anxiety)**	Moderate anxiety or greater	0.3129	0.1224	2.56	0.011
**Depression (Ref: No depression)**	Moderate depression or greater	0.1793	0.1167	1.54	0.125
**Network of potential living kidney donors (square root transformation)**		0.0071	0.01359	0.52	0.602
**Health literacy**		-0.02823	0.02385	-1.18	0.237
**Family loyalty**		-0.00088	0.04228	-0.02	0.983
**Religious objection (Ref: No religious objection to transplant)**	Any religious objection to transplant	0.04638	0.04598	1.01	0.313

© 2024 Vélez-Bermúdez M. *JAMA Network Open*.

eTable 12. Complete Output for Multivariable Regression Examining Race by Time, Trust in Physician

Effect		Estimate	Standard Error	t Value	Pr > |t|
**Intercept**		2.9107	0.2451	11.87	<.0001
**Race (Ref: White)**	Black	-0.04878	0.05274	-0.92	0.3553
**Time (Ref: Baseline)**	Follow-up	0.02609	0.02678	0.97	0.3302
**Race * Time**	Black * Follow-up	-0.1634	0.05361	-3.05	0.0024
**Age**		-0.00016	0.001643	-0.1	0.9209
**Gender (Ref: Male)**	Female	-0.03919	0.03813	-1.03	0.3044
**Marital status (Ref: Single)**	In a marital or domestic partnership	-0.04674	0.042	-1.11	0.2662
**Family income (Ref: less than $50K)**	$50K or Greater	0.06119	0.04841	1.26	0.2065
**Employment status (Ref: Unemployed)**	Part- or full-time	0.03025	0.04628	0.65	0.5135
**Transplant knowledge**		0.007961	0.007177	1.11	0.2677
**Number of learning activities**		-0.00888	0.02293	-0.39	0.6987
**Hours of learning activities (Ref: 0 – 2 hours)**	Greater than 2, less than or equal to 5 hours	-0.02173	0.05384	-0.4	0.6867
**Hours of learning activities (Ref: 0 – 2 hours)**	Greater than 5 hours	0.005496	0.05694	0.1	0.9231
**Transplant concerns**		0.001446	0.002462	0.59	0.5572
**Insurance status (Ref: Private only)**	Both Private and Public	-0.00499	0.0563	-0.09	0.9294
**Insurance status (Ref: Private only)**	Public only	0.04414	0.06072	0.73	0.4675
**Days from evaluation to Follow-up**		-0.00019	0.000127	-1.46	0.1443
**Waitlist status at Follow-up (Ref: Accepted for transplant)**	Evaluation closed or incomplete	-0.03202	0.04852	-0.66	0.5096
**Waitlist status at Follow-up (Ref: Accepted for transplant)**	Rejected for transplant	-0.046	0.05164	-0.89	0.3733
**Dialysis duration categorized (Ref: no dialysis)**	Greater than 0, less than 1 year	0.172	0.1183	1.45	0.1462
**Dialysis duration categorized (Ref: no dialysis)**	Greater than or equal to 1 year, less than 5 years	0.1809	0.1238	1.46	0.1442
**Dialysis duration categorized (Ref: no dialysis)**	Greater than or equal to 5 years	0.05706	0.1424	0.4	0.6887
**Dialysis modality (Ref: Not on dialysis)**	Hemodialysis	-0.2178	0.1166	-1.87	0.0622
**Dialysis modality (Ref: Not on dialysis)**	Peritoneal dialysis	-0.2039	0.1264	-1.61	0.1072

© 2024 Vélez-Bermúdez M. *JAMA Network Open*.

Effect		Estimate	Standard Error	t Value	Pr > |t|
**Social support**		0.0186	0.003125	5.95	<.0001
**Anxiety (Ref: No anxiety)**	Moderate anxiety or greater	-0.01113	0.0992	-0.11	0.9107
**Depression (Ref: No depression)**	Moderate depression or greater	-0.2242	0.09459	-2.37	0.018
**Network of potential living kidney donors (square root transformation)**		-0.00114	0.011	-0.1	0.9178
**Health literacy**		0.03492	0.01932	1.81	0.071
**Family loyalty**		0.06495	0.03418	1.9	0.0577
**Religious objection (Ref: No religious objection to transplant)**	Any religious objection to transplant	-0.08118	0.03722	-2.18	0.0295

© 2024 Vélez-Bermúdez M. *JAMA Network Open*.

eReferences

1. Murray LR, Conrad NE, Zarifian A. Perceptions of kidney transplant by persons with end stage renal disease/Research critique/Investigators' response. *Nephrol Nurs J*. 1999;26(5):479.
2. Waterman AD, Barrett AC, Stanley SL. Optimal transplant education for recipients to increase pursuit of living donation. *Prog Transpl*. 2008;18(1):55-62.
3. Waterman AD, Stanley SL, Covelli T, Hazel E, Hong BA, Brennan DC. Living Donation Decision Making: Recipients' Concerns and Educational Needs. *Prog Transplant*. 2006;16(1):7.
4. Charlson ME, Pompei P, Ales KL, MacKenzie CR. A new method of classifying prognostic comorbidity in longitudinal studies: development and validation. *J Chronic Dis*. 1987;40(5):373-383.
5. Bardis PD. A familism scale. *Marriage Fam Living*. Published online 1959.
6. Rumsey S, Hurford D, Cole A. Influence of knowledge and religiousness on attitudes toward organ donation. In: Vol 35. Elsevier; 2003:2845-2850.
7. Cohen S, Mermelstein R, Kamarck T, Hoberman HM. Measuring the functional components of social support. *Soc Support Theory Res Appl*. Published online 1985:73-94.
8. Derogatis LR. Brief symptom inventory. *Eur J Psychol Assess*. Published online 1975.
9. Bird ST, Bogart LM. Perceived race-based and socioeconomic status (SES)-based discrimination in interactions with health care providers. *Ethn Dis*. 2001;11(3):554-563.
10. Thorburn S, Lindly OJ. A systematic search and review of the discrimination in health care measure, and its adaptations. *Patient Educ Couns*. 2022;105(7):1703-1713.
11. LaVeist TA, Nickerson KJ, Bowie JV. Attitudes about racism, medical mistrust, and satisfaction with care among African American and white cardiac patients. *Med Care Res Rev*. 2000;57(1_suppl):146-161.
12. Boulware L, Ratner LE, Cooper LA, Sosa JA, LaVeist TA, Powe NR. Understanding disparities in donor behavior: race and gender differences in willingness to donate blood and cadaveric organs. *Med Care*. Published online 2002.
13. LaVeist TA, Isaac LA, Williams KP. Mistrust of health care organizations is associated with underutilization of health services. *Health Serv Res*. 2009;44(6):2093-2105.
14. Thom DH, Ribisl KM, Stewart AL, Luke DA, The Stanford Trust Study Physicians. Further validation and reliability testing of the Trust in Physician Scale. *Med Care*. Published online 1999:510-517.

© 2024 Vélez-Bermúdez M. *JAMA Network Open*.
