# Supplementary material for: Health Care Perceptions and a Concierge-Based Transplant Evaluation for Patients With Kidney Disease
Source: JAMA Netw Open. 2024 Nov 26;7(11):e2447335. doi: 10.1001/jamanetworkopen.2024.47335 (PMC11600232; doi:10.1001/jamanetworkopen.2024.47335)
Supplement: Supplement 2. — Data Sharing Statement [file jamanetwopen-e2447335-s002.pdf]

Data Sharing Statement

Vélez-Bermúdez. Health Care Perceptions and a Concierge-Based Transplant Evaluation for Patients With Kidney Disease. *JAMA Netw Open*. Published November 26, 2024. doi:10.1001/jamanetworkopen.2024.47335

Data
Data available: No

Additional Information
Explanation for why data not available: The data are not publicly available due to privacy or ethical restrictions.
